# Comparative clinical and epidemiological characteristics, risk factors, and outcomes of uterine torsion in camels, buffaloes, and cattle: a multicenter study

**DOI:** 10.3389/fvets.2026.1790208

**Published:** 2026-03-04

**Authors:** Ahmed Ali, Derar R. Derar, Mohamed Rawy, Yousef M. Alharbi

**Affiliations:** 1 Department of Clinical Sciences, College of Veterinary Medicine, Qassim University, Buraydah, Saudi Arabia; 2 Department of Theriogenology, Faculty of Veterinary Medicine, Minia University, Minya, Egypt; 3 Department of Medical Biosciences, College of Veterinary Medicine, Qassim University, Buraydah, Saudi Arabia

**Keywords:** buffalo, camel, cattle, clinical features, prevalence, risk factors, species comparison, uterine torsion

## Abstract

**Background:**

Uterine torsion is a leading cause of dystocia in large domestic animals. However, there is no systematic comparison of its clinical presentation, epidemiological data, risk factors, or outcomes in camelids, buffalo, and cattle.

**Methods:**

Between 2008 and 2023, data were collected from closed and open housing systems in multiple regions (Qassim region, Saudi Arabia and Minia Governorate, Egypt). A total of 3,557 camels (*Camelus dromedarius*), 7,157 buffaloes (*Bubalus bubalis*), and 5,251 cattle (*Bos indicus*) were examined. Detailed clinical records from 80, 262, and 129 diagnosed torsion cases were examined for parity, torsion characteristics, intervention methods, and survival outcomes. Logistic regression models were used to determine risk factors for fetal and maternal mortality.

**Results:**

Closed housing led to significantly higher rates of uterine torsion in all species (*p* < 0.001), with multiparous females being more likely to experience it. Gestational timing, torsion direction, anatomical location, and severity all differed significantly between species. Torsion occurred primarily during parturition in camels and cattle, but was evenly distributed between late gestation and parturition in buffaloes. Despite left-horn pregnancies, camels exhibited almost exclusively right-sided torsion, whereas buffaloes and cattle exhibited torsion in the direction of the gravid horn. Camels (96.3%) and buffaloes (98.6%) had more post-cervical torsion, whereas cattle had a higher rate of pre-cervical torsion (35.7%). Camels had the highest percentage of severe torsion (85.0%), buffaloes had severe (45.8%) and moderate (48.9%) torsions, and cattle had moderate (67.4%) to mild (24.8%) torsions. Severe torsion and delayed intervention (>48 h) were the strongest risk factors for fetal and maternal mortality across all species.

**Conclusion:**

This study reveals significant species-specific differences in the clinical presentation and epidemiology of uterine torsion. The identified patterns in timing, direction, location, and severity provide critical evidence for developing tailored diagnostic and management strategies in camels, buffaloes, and cattle.

## Graphical abstract

**Figure:**
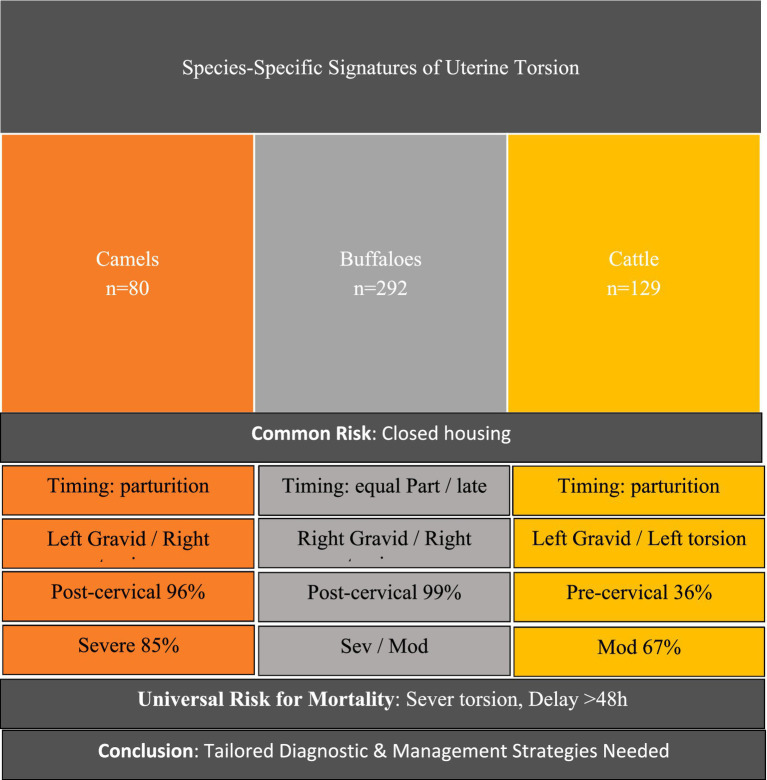
A graphical summary of the study. This illustration synthesizes key findings from a comparative study (2008–2023) of uterine torsion in 3557 camels, 7,157 buffaloes, and 5,251 cattle. It contrasts species-specific patterns in: (1) Risk and timing: the universal risk of closed housing and differences in onset (parturition vs. late gestation); (2) Torsion characteristics: direction relative to the gravid horn, anatomical location (pre- vs. post-cervical), and dominant severity; and (3) Outcomes: identifying severe torsion and delayed intervention (>48 h) as the strongest universal risk factors for mortality. The data underscore the need for tailored clinical management across species.

## 1 Introduction

Uterine torsion, defined as the rotation of the gravid uterus around its longitudinal axis, is a major obstetrical emergency in large domestic animals and is a known cause of dystocia, fetal mortality, and maternal loss (1–6). The condition can cause partial or complete obstruction of the birth canal and is frequently associated with impaired uterine perfusion (1–3, 7–9). Reported complications include fetal death, premature delivery, fetal membrane retention, uterine rupture, endotoxemia, infertility, and, in severe or prolonged cases, maternal death (1–4, 6, 7, 10, 11, 42).

The clinical presentation, diagnostic approach, and management of uterine torsion differ significantly across species. Clinical signs in camels can range from subtle behavioral changes to severe abdominal discomfort and colic, resulting in delayed diagnosis (1, 6, 12). Buffaloes frequently exhibit prolonged straining, decreased feed intake, and constipation (5, 9, 10, 13, 14), whereas cattle may show systemic signs such as fever, tachycardia, and anorexia (3, 8, 15–19).

A combination of vaginal and rectal examinations is typically used to make the diagnosis, which allows for the identification of spiral vaginal folds, the assessment of broad ligament tension, and the determination of the direction and degree of torsion (1, 3, 7, 8, 12, 18, 20).

Therapeutic options include manual or plank-rolling detorsion and cesarean section, with the choice of intervention guided by torsion severity, duration of dystocia, cervical status, and the condition of the mother and fetus (7, 9, 12, 20–25).

Although uterine torsion is a well-known condition in large animal practice, most published research focuses on a single species, typically cattle, and is frequently limited to specific clinical settings. As a result, comparative data on species-specific differences in epidemiological patterns, clinical presentation, management, and outcomes among camels, buffaloes, and cattle is limited, particularly for camels, despite their growing economic importance and unique obstetrical challenges.

The aim of this multicenter study was to compare the clinical and epidemiological characteristics of uterine torsion in camels, buffaloes, and cattle, including differences in prevalence, housing system, parity, gestational timing, torsion features, management strategies, and maternal and fetal outcomes, as well as to identify factors associated with fetal and maternal mortality to aid in species-appropriate obstetric decision-making.

## 2 Materials and methods

### 2.1 Study design, population, and data collection

This was designed as a multicenter study. Data was obtained from veterinary clinical records and farm surveillance at several sites, including privately owned farms and referral veterinary clinics in the Qassim region of central Saudi Arabia and Minia Governorate in Upper Egypt, both of which are major livestock-producing areas with diverse management practices. The use of multiple centers was intended to increase the sample size and improve the generalizability of the findings across various management systems and geographical regions. Data was collected using standardized diagnostic and recording protocols at all participating sites.

Animals were divided into two housing systems: open system, which is defined as pasture-based management with free movement and minimal confinement. Closed system is defined as housed management with limited space and movement, such as tie-stall or loose-housing barns.

The study population consisted of uterine torsion cases identified from the total number of parturitions (calvings) recorded for each species during the study period at the participating centers. According to farm birth logs, a total of 3,557 camels, 7,157 buffaloes, and 5,251 cattle calved during the study period. These parturitions resulted in 80, 262, and 129 cases of uterine torsion, respectively. The study was carried out over a 15-year period (2008–2023).

To determine the proportion of uterine torsion among dystocia cases, data from referral clinics were analyzed: camels (*n* = 192 dystocia cases), buffaloes (*n* = 161), and cattle (*n* = 312).

Across all participating centers and throughout the study period, uterine torsion cases were diagnosed and recorded using uniform clinical definitions and obstetrical criteria derived from standard veterinary textbooks (18, 26). A predefined case record form was used to document species, parity, housing system, gestational stage, torsion direction, torsion degree, cervical involvement, maternal condition at presentation, method of correction, fetal viability, and maternal outcome. Only cases with complete diagnostic and outcome data were included in the final analysis.

### 2.2 Diagnostic and obstetrical examination

Uterine torsion was diagnosed using a combination of clinical history, rectal palpation, and vaginal examination performed by experienced veterinarians following established obstetrical procedures (18, 26). Each case underwent a standardized obstetrical examination, which included a vaginal examination to detect spiral folds indicating torsion direction and determining whether the torsion was pre-cervical (cervix accessible) or post-cervical (cervix not reachable). (2) Rectal palpation to confirm the diagnosis, measure tension in the broad ligaments, and identify the pregnant horn (right or left). (3) Torsion degree is classified as mild (45–90°), moderate (>90–180°), or severe (>180°) using established methods that combine vaginal and rectal findings (18, 26). (4) Torsion direction, indicated as clockwise (right-sided) or counterclockwise (left-sided) from the veterinarian’s perspective. (5) Maternal health status at presentation, classified as “good” (alert, good appetite), “fair” (reduced appetite and response), or “poor” (recumbent, off feed, dull).

### 2.3 Intervention and outcome documentation

Therapeutic management was determined case-by-case, taking into account species, degree of torsion, cervical status, duration of dystocia, and maternal condition, in accordance with standard large-animal obstetrical guidelines (18, 26). The attending veterinarian chose the following correction method: (1) Rolling Method, with the dam in lateral recumbency on the torsion side. A plank was placed on the flank, and the animal was gently rolled in the direction of torsion. (2) Cesarean section performed with local infiltration analgesia (2% lidocaine, 10 mg/kg) via flank approach in standing cattle/buffaloes or left ventrolateral approach in camels in sternal recumbency (1, 2, 12, 22, 27, 28). Cesarean section was chosen when manual or rolling correction failed or was contraindicated, particularly in cases of advanced torsion, compromised maternal condition, or a lack of cervical dilation. Following the intervention, animals were subjected to intensive postoperative monitoring, which included fluid therapy, antimicrobial treatment, anti-inflammatory medication, and supportive care as needed.

The following outcomes were recorded: successful detorsion, fetal viability (alive/dead at intervention), and maternal survival. Maternal survival was defined as the dam remaining alive 7 days after intervention. This brief survival period is consistent with the outcome assessment windows used in major clinical studies of uterine torsion to capture complications directly related to the condition or its surgical treatment (3, 6, 7, 9, 13, 25). The time from first observed signs to intervention was classified as early (<24 h), moderate (24–48 h), or late (>48 h).

### 2.4 Statistical analysis

The data were analyzed with SPSS software (version 15, IBM Corp.). Differences in proportions (for example, prevalence between housing systems, species differences in torsion location) were tested using the Chi-square or Fisher’s exact test, as appropriate. *p*-values were reported to three decimal places, with *p* < 0.05 indicating significance.

Separate binary logistic regression models were developed for camels, buffaloes, and cattle to identify risk factors for fetal and maternal mortality. A pooled model with all species was also examined. Torsion severity, time to intervention, gestation stage (late gestation vs. parturition), parity (primiparous vs. multiparous), torsion location (pre- or post-cervical), and maternal health status were all predictive variables. The results are expressed as odds ratios [Exp(B)] with 95% confidence intervals.

## 3 Results

### 3.1 Prevalence and influence of housing system

**Figure 1:**
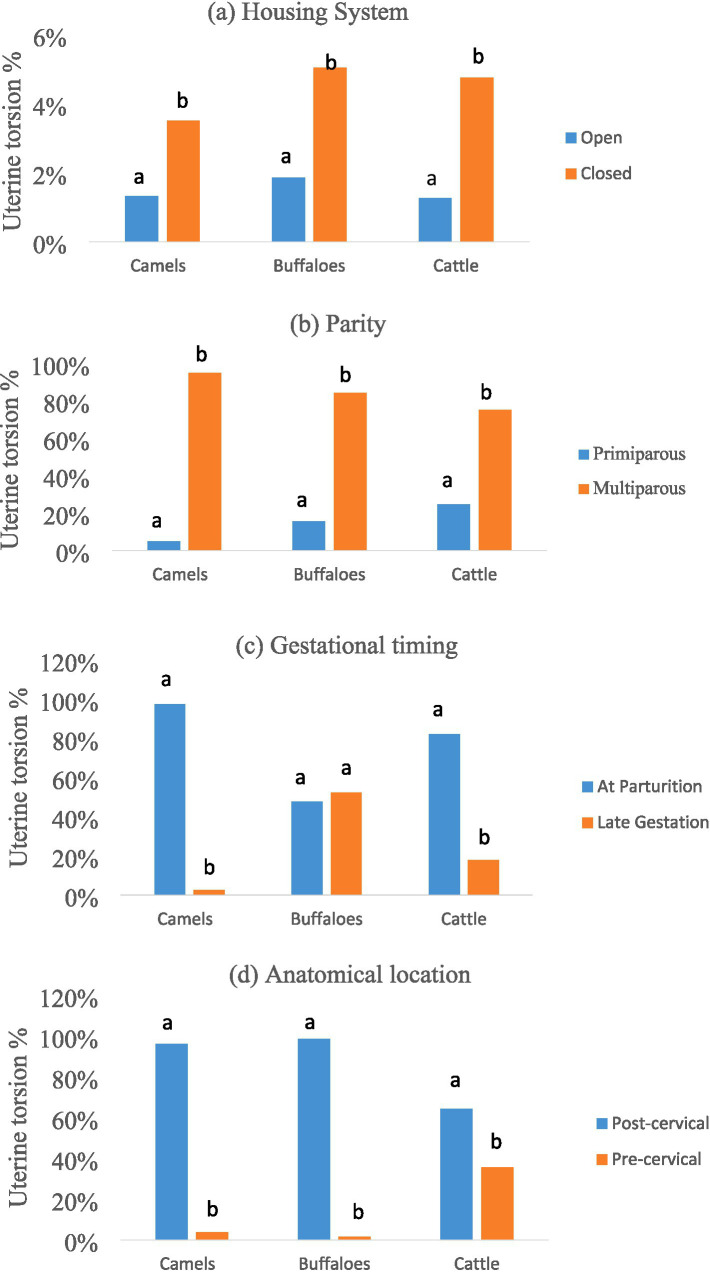
Species-specific patterns of uterine torsion in camels, buffaloes, and cattle. **(a)** Influence of housing system on the prevalence of uterine torsion, showing significantly higher rates under closed housing compared with open housing within each species (*p* < 0.001). **(b)** Association between parity and uterine torsion, with significantly higher occurrence in multiparous than primiparous animals across species (*p* < 0.001). **(c)** Timing of uterine torsion onset, demonstrating species differences between occurrence at parturition and late gestation (*p* < 0.001). **(d)** Anatomical location of uterine torsion, illustrating significant interspecies variation between post-cervical and pre-cervical torsion (*p* < 0.001). Different letters (a, b) indicate significant differences within each species. Sample sizes were 3,557 camels, 7,157 buffaloes, and 5,251 cattle for prevalence analysis, and 80 camels, 262 buffaloes, and 129 cattle for torsion-characteristic analyses.

The overall prevalence of uterine torsion was 2.95% (471/15,965). Closed housing had a significantly higher prevalence (4.66%) than open housing (1.50%) across all species (*p* < 0.001; see Figure 1a).

Within closed housing, buffaloes had a significantly higher incidence than camels (*p* = 0.015), but there were no significant differences between buffaloes and cattle (*p* = 0.695), camels and cattle (*p* = 0.081) or camels and buffaloes (*p* = 0.072). Under open housing, there were no significant differences between species (*p* = 0.108).

Uterine torsion caused 19.27% (37/192) of dystocia cases in camels, 8.12% (69/161) in buffaloes, and 7.69% (24/312) in cattle, indicating a significant difference between species (χ^2^ = 83.51, *p* < 0.001).

### 3.2 Association with parity

Uterine torsion was significantly more common in multiparous animals across all species (*p* < 0.001; Figure 1b).

### 3.3 Gestational timing

Torsion onset varied by species (Figure 1c). Torsion was most common in camels and cattle during parturition (*p* < 0.001). Torsion in buffaloes was nearly evenly distributed between parturition and late gestation, with no significant difference (*p* = 0.294).

### 3.4 Anatomical location of torsion

Post-cervical torsion was significantly more frequent in camels (96.3%) and buffaloes (98.6%), whereas cattle showed a significantly higher proportion of pre-cervical torsion (35.7%; *p* < 0.001; Figure 1d).

### 3.5 Association between pregnant horn and torsion direction

A clear species-specific pattern was found (Table 1). Torsion was almost exclusively right-sided in camels (97.5%), despite all pregnancies occurring in the left horn (*p* = 1.0, indicating no association). In contrast, in buffaloes and cattle, torsion direction was significantly associated with the side of the gravid horn (*p* < 0.001), occurring predominantly on that side.

**Table 1:** Association between pregnant horn and torsion direction.

Species	Torsion direction	Pregnant horn right (*n*, %)	Pregnant horn left (*n*, %)	Total	*p*-value
Camels	Right	0 (0.0%)	78 (97.5%)	78	1.0
	Left	0 (0.0%)	2 (2.5%)	2	
Buffaloes	Right	194 (74.0%)	55 (21.0%)	249	<0.001
	Left	0 (0.0%)	13 (5.0%)	13	
Cattle	Right	70 (54.3%)	8 (6.2%)	78	<0.001
	Left	18 (14.0%)	33 (25.6%)	51	

### 3.6 Association between species and torsion severity

Torsion severity was significantly associated with species (χ^2^ = 265.3, *p* < 0.001; Table 2). Camels exhibited predominantly severe torsion (>180°; 85.0%), buffaloes had an even distribution of severe (45.8%) and moderate (48.9%) cases, and cattle primarily had moderate (67.4%) or mild (24.8%) torsions.

**Table 2:** Species differences in torsion severity.

Species	Severe (>180°)	Moderate (>90–180°)	Mild (45–90°)	Total *n*
Camels	68 (85.0%)a	3 (3.8%)a	9 (11.3%)a	80
Buffaloes	120 (45.8%)b	128 (48.9%)b	14 (5.3%)a	262
Cattle	10 (7.8%)c	87 (67.4%)c	32 (24.8%)b	129
Total	198 (42.0%)	224 (47.6%)	49 (10.4%)	471

Different letters within the same column indicate significant differences between species (*p* < 0.05).

### 3.7 Risk factors for fetal and maternal mortality

Logistic regression analyses revealed consistent risk factors across species (Tables 3, 4). The strongest predictors of fetal mortality were severe torsion and longer time to intervention. Significant risk factors for maternal mortality included fetal death, severe torsion, delayed intervention (more than 48 h), and the use of a cesarean section.

**Table 3:** Logistic regression analysis of factors affecting fetal mortality in uterine torsion cases.

Predictor	Camel B (*p*-value)/OR [95% CI]	Buffalo B (*p*-value)/OR [95% CI]	Cattle B (*p*-value)/OR [95% CI]	Pooled model B (*p-*value)/OR [95% CI]
Severity	1.72 (<0.001)/5.60 [3.1–10.1]	1.58 (0.004)/4.85 [1.6–9.0]	1.41 (<0.001)/4.10 [2.0–8.3]	1.56 (<0.001)/4.74 [3.2–7.0]
Time until intervention	1.35 (0.003)/3.86 [1.5–9.8]	1.12 (0.030)/3.07 [1.1–8.5]	1.18 (0.002)/3.26 [1.4–7.8]	1.23 (<0.001)/3.41 [2.2–5.3]
Gestation period	0.20 (0.600)/1.22 [0.6–2.5]	0.28 (0.500)/1.32 [0.5–3.4]	0.12 (0.720)/1.13 [0.5–2.6]	0.18 (0.580)/1.19 [0.8–1.8]
Maternal health	0.88 (0.020)/2.41 [1.2–4.8]	0.65 (0.120)/1.92 [0.8–4.5]	0.79 (0.010)/2.20 [1.2–3.9]	0.81 (<0.001)/2.25 [1.5–3.4]
Parity	0.10 (0.700)/1.11 [0.7–1.7]	0.05 (0.850)/1.05 [0.6–1.8]	0.15 (0.650)/1.16 [0.7–1.9]	0.11 (0.680)/1.12 [0.9–1.4]
Location	0.12 (0.800)/1.13 [0.6–2.1]	0.09 (0.880)/1.10 [0.5–2.3]	0.10 (0.810)/1.10 [0.6–2.0]	0.11 (0.770)/1.12 [0.8–1.5]

B, regression coefficient; OR, odds ratio; CI, confidence interval.

**Table 4:** Logistic regression analysis of risk factors for maternal mortality.

Predictor	Camel B (*p*-value)/OR [95% CI]	Buffalo B (*p*-value)/OR [95% CI]	Cattle B (*p*-value)/OR [95% CI]	Pooled model B (*p*-value)/OR [95% CI]
Fetal viability (dead)	1.85 (0.032)/6.36 [1.17–34.6]	1.20 (0.048)/3.32 [1.01–10.9]	1.42 (0.040)/4.14 [1.05–16.3]	1.52 (<0.001)/4.58 [2.0–10.5]
Severity (moderate/severe)	2.10 (0.015)/8.16 [1.53–43.6]	1.45 (0.020)/4.27 [1.26–14.5]	1.75 (0.022)/5.75 [1.29–25.7]	1.85 (<0.001)/6.36 [2.5–16.1]
Parity (multipara)	0.65 (0.210)/1.91 [0.71–5.12]	0.40 (0.345)/1.49 [0.64–3.48]	0.52 (0.310)/1.68 [0.65–4.36]	0.55 (0.040)/1.73 [1.02–2.93]
Method (cesarean)	1.20 (0.045)/3.32 [1.03–10.7]	0.88 (0.120)/2.41 [0.81–7.15]	1.00 (0.050)/2.71 [1.0–7.35]	1.05 (0.010)/2.85 [1.28–6.33]
Gestation (late)	0.80 (0.125)/2.22 [0.83–5.92]	0.65 (0.210)/1.91 [0.69–5.28]	0.72 (0.180)/2.05 [0.73–5.76]	0.75 (0.035)/2.12 [1.05–4.26]
Time to interference (>48 h)	1.60 (0.005)/4.95 [1.63–15.0]	1.42 (0.015)/4.14 [1.32–13.0]	1.50 (0.010)/4.48 [1.44–13.9]	1.55 (<0.001)/4.71 [2.0–11.1]

B, regression coefficient; OR, odds ratio; CI, confidence interval.

## 4 Discussion

This multicenter study presents a detailed comparative analysis and epidemiological overview of uterine torsion in three major domestic species: camels, buffaloes, and cattle. By focusing on clinical features, management, and outcomes, we discovered distinct species-specific patterns with direct implications for veterinary practice.

The most notable findings of this study are the significant interspecies differences in clinical presentation. In buffaloes, torsion is evenly distributed between late gestation and parturition, whereas camels and cattle have a clear parturient onset. This could be linked to behavioral differences in buffaloes, such as frequent wallowing and postural changes (29, 30), which could exert torsional forces on the gravid uterus prior to the onset of labor.

The higher prevalence of uterine torsion in closed housing systems across all species is consistent with previous reports (1, 2, 18, 30–33), supporting the hypothesis that restricted movement contributes to abdominal laxity and predisposes to uterine displacement. The particularly high incidence in closed-managed buffaloes calls for further research into breed-specific anatomy and management practices. In open systems, where animals have more freedom of movement, the prevalence of uterine torsion was uniformly lower and did not vary significantly by species.

The consistent right-sided torsion in camels, despite invariable left-horn pregnancy, highlights a unique anatomical constraint, most likely the rumen position (33–36). The overwhelming predominance of post-cervical torsion in camels and buffaloes, as opposed to the notable rate of pre-cervical torsion in cattle, is likely due to differences in uterine suspension, ligament elasticity, and fetal size (4, 8, 16, 17, 32, 37).

Camels had the most severe torsions, which could be explained by a less elastic uterine structure and a fetus confined to a single horn (33, 34, 36, 38, 39). The milder presentations in cattle may allow for earlier diagnosis and successful manual correction. Furthermore, uterine torsion caused a greater proportion of dystocia cases in camels than in buffaloes or cattle. This finding is consistent with previous research, which shows that dystocia in camels is frequently detected at advanced stages, particularly in open or semi-open production systems (1). In contrast, closer calving supervision in cattle and buffaloes may allow earlier detection of obstetrical problems and reduce progression to severe torsion (5, 9, 28, 40).

Parity was consistently linked to uterine torsion across all species, with multiparous animals being more affected than primiparous ones. This finding supports previous reports in cattle and buffaloes (5, 10, 11, 31) and could be linked to reduced uterine tone, increased uterine volume, or prior stretching of supporting structures in multiparous dams, though causal mechanisms cannot be inferred from the current data.

Management strategies differed by species. Rolling techniques were more successful in cattle, especially in cases of moderate torsion and adequate cervical dilation (7, 16–18, 24). Cesarean section was more commonly performed in camels, most likely due to more severe torsion, delayed presentation, and anatomical constraints such as the hump, which limit nonsurgical correction (1, 12). Buffaloes followed an intermediate pattern, with both rolling and surgical interventions used depending on case severity (13, 21, 23, 41). Overall, these findings highlight the importance of species-specific management rather than a standardized approach to uterine torsion.

Our regression models confirm that the degree of torsion and the timing of intervention are the most important predictors of survival for both the dam and the fetus, in line with previous research (3, 13, 17, 23). The link between cesarean section and higher maternal mortality is likely due to a selection bias, as surgical intervention is typically reserved for the most severe, protracted, or complicated cases in which the dam’s condition is already critical (1).

This study has limitations due to variability in farm management, clinician experience, and record completeness, which could have influenced case detection and outcomes. Nonetheless, the multicenter design, large sample size, and extended study period provide a reliable representation of uterine torsion seen in routine veterinary practice. There were no detailed data on total births per season for seasonal species, so seasonal incidence could only be estimated. Furthermore, while the definitions of “closed” and “open” systems have been clarified, they may still encompass a variety of practices.

## 5 Conclusion

This study identifies clear species-specific profiles of uterine torsion in camels, buffaloes, and cattle. There are significant differences in the timing, direction, anatomical location, and typical severity of torsion. Awareness of these differences can lead to earlier diagnosis, appropriate intervention, and better outcomes, especially in camelid practice, where delayed presentation remains a significant challenge. These clinical distinctions, combined with the universally negative impact of severe torsion and treatment delay on survival, highlight the need for species-specific diagnostic protocols and the critical importance of timely veterinary intervention. Future prospective research should seek to quantify the specific biomechanical and management risk factors identified by these observational findings.

## Data Availability

The original contributions presented in the study are included in the article/supplementary material, further inquiries can be directed to the corresponding author.
